# Personal

**Published:** 1906-08

**Authors:** 


### PERSONAL.

Dr. Edwin A. Bowerman, 234 East Utica Street, was reelected secretary of the Buffalo Academy of Medicine at its last annual meeting.

Dr. A. C. Griffith, of Kansas City, recently paid a visit to Buffalo for a few hours. Dr. Griffith and Mr. Watson Armour, vice-president of Armour & Co., and in charge of the Kansas City works, are making a little vacation tour of the east, their objective point being the White Mountains.

Dr. Nelson W. Wilson, of Buffalo, has been appointed lecturer on diseases of the genitourinary system at the University of Buffalo, vice Dr. D. W. Harrington, deceased. Dr. Wilson has special fitness for the duty to which he has been assigned, having pursued studies and limited his practice for some years to that department of medicine.

Dr. Julius Pohlman, of Buffalo, who, with his family, has been in Europe for some weeks, has returned and resumed the practice of his specialty—ophthalmology.

Dr. L. A. Whitney, for the past several years resident physician and surgeon and assistant superintendent of the New York State Hospital for Crippled and Deformed Children, at West Haverstraw, N. Y., announces his resignation from that institution and location at 33 Clinton Avenue, South, Rochester, N. Y., for the special practice of orthopedic surgery.

